# The ROCK-ezrin signaling pathway mediates LPS-induced cytokine production in pulmonary alveolar epithelial cells

**DOI:** 10.1186/s12964-022-00879-3

**Published:** 2022-05-12

**Authors:** Ning Ding, Pibao Li, Huiqing Li, Yunlong Lei, Zengzhen Zhang

**Affiliations:** 1 Department of Anesthesiology, Shandong Provincial Third Hospital, Cheeloo College of Medicine, Shandong University, Jinan, 250031 Shandong China; 2 Department of Intensive Care Unit, Shandong Provincial Third Hospital, Cheeloo College of Medicine, Shandong University, Jinan, 250031 China

**Keywords:** ROCK, Ezrin, LPS, Signal pathway, Inflammation

## Abstract

**Background:**

Ezrin/radixin/moesin (ERM) proteins are members of the protein 4.1 superfamily and function as linkers that connect the actin cytoskeleton to the plasma membrane of cells. ERM also play critical role in the Lipopolysaccharide (LPS)-induced inflammatory response. However, the signaling mechanisms involved in this process remain unclear. In this study, we aimed to investigate the potential role of the rho-associated coiled-coil containing protein kinase (ROCK) pathway in LPS-induced ezrin phosphorylation and cytokine production in pulmonary alveolar epithelial cells.

**Methods:**

Cultured A549 and HPAEpiC cells were treated with LPS. The expression and localization of ezrin in A549 and HPAEpiC cells were then analyzed by western blotting and immunoflurescence. Activation of RhoA/ROCK was assessed by western blotting and RhoA activity assays. The interaction of ezrin with Syk and myeloid differentiation factor 88 (MyD88)/IL-1R-associated kinase 1 (IRAK-1) was investigated by co-immunoprecipitation. The activation of nuclear factor-κB (NF-κB) and mitogen-activated protein kinase (MAPK) was measured with electrophoretic mobility shift assays and by western blotting. ELISA and western blotting were performed to detect the levels of tumor necrosis factor-α (TNF-α), interleukin-1β (IL-1β), and high mobility group box 1 protein (HMGB1) release into the culture supernatant, and cellular HMGB1 levels.

**Results:**

LPS induced ezrin phosphorylation in a concentration- and time-dependent manner. The blockade of RhoA/ROCK inhibited LPS-induced ezrin phosphorylation and its translocation from the cytoplasm to the cell membrane. Co-immunoprecipitation assays further revealed that ezrin associated with Syk constitutively, but only associated with MyD88/IRAK-1 upon LPS challenge. Moreover, LPS-induced p38 and nuclear NF-κB activation was found to be ezrin dependent. The suppression of ezrin by siRNA or the blockade of ROCK activation with Y-27632 reduced the production of TNF-α, IL-1β, and HMGB1 in response to LPS.

**Conclusions:**

Our findings reveal a novel regulatory mechanism involving ezrin in the LPS-induced production of pro-inflammatory cytokines, and highlight the importance of the RhoA/ROCK-ezrin/Syk-MyD88/IRAK1 axis. Data presented in this manuscript provide novel insights into the signaling pathways activated in pulmonary alveolar epithelial cells by LPS.

**Video Abstract**

**Supplementary Information:**

The online version contains supplementary material available at 10.1186/s12964-022-00879-3.

## Background

Acute lung injury (ALI)/acute respiratory distress syndrome (ARDS) is a clinical syndrome that involves persistent lung inflammation caused by various direct and indirect stimuli. This condition affects approximately three million patients annually, and accounts for 10% of intensive care unit (ICU) admissions [1]. Although significant progress has been made in the treatment of ALI/ARDS, the mortality rate remains as high as 40% [2, 3]. One of the most critical causes of ALI/ARDS is bacterial sepsis with either a pulmonary or non-pulmonary source [4]. Lipopolysaccharide (LPS), the endotoxin portion of the gram-negative bacteria cell wall, induces lung inflammatory response and has been proven to be an important factor that can lead to ALI/ARDS [5].

It is well known that LPS binds toll-like receptor 4 (TLR4) and stimulates both mitogen-activated protein kinase (MAPK) and nuclear factor-κB (NF-κB) to induce the synthesis and release of inflammatory cytokines. Ezrin, a plasma membrane-actin cytoskeleton crosslinking protein of the Ezrin-radixin-moesin (ERM) family, has recently been reported to participate in the activation of TLR4 signaling when challenged by LPS [6]. Phosphorylation of the conserved T567 residue of ezrin resulted in the loss of endothelial barrier integrity, leading to lung inflammation [7, 8]. Interestingly, ezrin has been reported to regulate the production of cytokine interleukin-10 (IL-10) in B cells upon LPS stimulation [9]. Previous studies demonstrated that ezrin is not only involved in cytoskeletal organization but also represents a platform for the transmission of signals in response to LPS through its ability to cross-link transmembrane receptors with downstream signaling components [10]. Rho-associated coiled-coil containing protein kinase (ROCK) is known to regulate the cross-linking activities of ERM and mediate phosphorylation of the C-terminal domain of ERM proteins to stabilize their active state [11]. Inhibition of ROCK activity was previously shown to improve endothelial permeability and alleviate inflammatory reaction in a sepsis-induced rat model of ALI [12]. Therefore, we hypothesized that the RhoA/ROCK pathway plays a vital role in the activation of ezrin in LPS-induced inflammatory response.

In the present study, we tested whether ezrin is activated via the RhoA/ROCK pathway and investigated potential signal transduction mechanisms involved in the response to LPS. The results derived from this study will not only improve our understanding of the mechanisms associated with the action of ezrin in regulating the inflammatory reaction in pulmonary alveolar epithelial cells, but also offers an opportunity to target ezrin when developing novel therapies for the management of ALI/ARDS.

## Material and methods

### Cell culture and treatment

A549 cells and the HPAEpiC cell line was obtained from the American Type Culture Collection (Manassas, VA, USA). Cells were grown in Dulbecco's Modified Eagle Medium (DMEM) supplemented with 10% fetal bovine serum and antibiotic/antimycotic. For dose–response experiments, cells were treated with LPS (*Escherichia coli*, serotype O111:B4, Sigma, St. Louis, MO, USA) at concentrations of 0.1, 1 and 10 µg/mL for 3 h. For time-course experiments, cells were treated with 1 µg/mL of LPS for 0.5, 1, 3, 6 and 12 h. In ROCK blockade experiments, cells were pretreated with Y-27632 (Sigma, St. Louis, MO, USA) at a concentration of 50 µmol/L for 1 h before being exposed to LPS (1 µg/mL). Cells were seeded in 35-mm petri dishes at a density of 5 × 10^4^/mL for immunofluorescence and 5 × 10^5^/mL for western blotting and ELISA.

### Western blotting

After treatment, cells were washed with ice-cold phosphate buffered saline (PBS) and then exposed to radio immunoprecipitation assay (RIPA) lysis buffer [supplemented with 1 mmol/L phenylmethylsulfonyl fluoride (PMSF), and 1 mmol/L phosphatase inhibitor cocktail (PIC)]. A bicinchoninic acid (BCA) protein analysis kit (Pierce, Rockford, IL, USA) was used to quantify protein concentration. Equal amounts of protein were resolved by sodium dodecyl sulfate polyacrylamide gel electrophoresis (SDS-PAGE) and then transferred to 0.45 µm polyvinyl difluoride (PVDF) membranes. Membranes were blocked with 5% bovine serum albumin (BSA) diluted in tris-buffered saline containing Tween 20 (TBST) for 1 h and then incubated with primary antibodies against p38 (1:1000), p-p38 (1:2000), ERK1/2 (1:1000), p-ERK1/2 (1:1000), JNK (1:1000), p-JNK (1:500) (all from Cell Signaling, Danvers, MA, USA); and ezrin (1:1000), p-ezrin (1:1000), ROCK1 (1:1000), IKKβ (1:2000), p-IKKβ (1:500), IκBα (1:2000), p-IκBα (1:1000) and high mobility group box 1 protein (HMGB1, 1:1000) (all from Abcam, Cambridge, MA, USA). β-actin was used as internal standard. Following incubation, PVDF membranes were washed with TBST and then incubated with peroxidase-conjugated specific secondary antibody for 1 h at room temperature. Bands were visualized by chemiluminescence, representative images were acquired, and Image J software (NIH Image, Bethesda, MD, USA) was used to quantify the density of each band.

### Flow cytometry

A549 and HPAEpiC cells were fixed with 4% paraformaldehyde (pH 7.4) for 30 min, permeabilized with 0.3% Triton X-100, incubated with p-ezrin antibody (1:1000, Invitrogen, Carlsbad, CA, USA) 2 h at 37 °C, and then incubated with a fluorescent second antibody for 1 h at 37 °C. After incubation, cells were washed in washing solution (PBS containing 0.1% sodium azide and 1% fetal bovine serum) and centrifuged. The pellet was resuspended in rhodamine-conjugated phalloidin and read on a BD LSRII flow cytometer (BD Biosciences, Franklin Lakes, NJ, USA) and analyzed using FlowJo software (Treestar, Ashland, OR, USA).

### RhoA activity assays

Active GTP-bound RhoA was detected using lysates collected from A549 and HPAEpiC cells using a RhoA activation assay kit (Abcam, Cambridge, MA, USA); this kit was used in accordance with the manufacturer’s indications. In brief, cell lysates were centrifuged at 13,000 × *g* at 4 °C for 3 min, and equal volumes of lysates were treated with rhotekin-Rho-binding domain-coated agarose beads at 4 °C for 1 h. Bead-precipitated proteins were then fractionated and immunoblotted with an antibody against RhoA (1:1000, Abcam, Cambridge, MA, USA).

### Co-immunoprecipitation

Whole cell lysates were incubated overnight with anti-ezrin antibody (1:1000). Immune complexes were precipitated with protein A/G agarose beads for 6 h and then washed three times with immunoprecipitation buffer. Immunoprecipitated proteins were then eluted with 2 × SDS loading buffer and analyzed by western blotting using anti-Syk (1:2000), anti-myeloid differentiation factor 88 (MyD88, 1:1000), anti-IL-1R-associated kinase 1 (IRAK-1, 1:1000), or anti-ezrin (1:1000) (all from Abcam, Cambridge, MA, USA), as described above.

### Fluorescence microscopy

Cells were washed with PBS and fixed with 4% paraformaldehyde for 30 min at room temperature. Cells were then permeabilized with 0.5% Triton X-100 for 10 min and blocked with 5% BSA in TBST for 1 h. The samples were then incubated with anti-p-ezrin antibody (1:500, Abcam, Cambridge, MA, USA) in TBST overnight at 4 °C. The cells were washed with TBST and incubated with Alexa Fluor 488-conjugated AffiniPure goat anti-rabbit IgG (1:200, ZSGB-BIO, Beijing, China) and Rhodamine-phalloidin (Molecular Probes, Carlsbad, CA, USA) to stain p-ezrin and F-actin, respectively. Nuclei were stained with DAPI (Invitrogen, Carlsbad, CA, USA). Samples were washed three times with TBST prior to confocal microscopy (Olympus FV1000, Olympus, Tokyo, Japan).

### siRNA transfection

For transient knockdown experiments, cells were transfected with ROCK1-specific siRNA (ROCK1 siRNA) or control siRNA, and ezrin specific siRNA (ezrin siRNA) or control siRNA (all from Santa Cruz Biotechnology, Santa Cruz, CA, USA), respectively. Cells were first plated onto 6-well plates. ROCK1 siRNA, ezrin siRNA or control siRNA duplexes were diluted to a final concentration of 10 μmol/L in OptiMem (Invitrogen, San Diego, CA, USA) and incubated with Lipofectamine 3000 transfection reagent (Thermo Fisher Scientific, Waltham, MA, USA) at room temperature for 15 min. The mixture was then incubated with the cells under serum- and antibiotic-free conditions for 18 h at 37 °C. Cells were then washed twice with sterile PBS and incubated in William's E medium supplemented with 5% calf serum for 24 h prior to exposure to LPS.

### Reverse transcription PCR and quantitative real-time PCR

RNA was extracted from A549 cells using the RNeasy Mini Kit (Qiagen, Valencia, CA, USA). An iScript reverse transcription supermix kit (Bio-Rad, Hercules, CA, USA) was used for reverse transcription. PCR amplification mixtures were prepared using iTaq™ Fast SYBR Green Supermix with ROX (Bio-Rad, Hercules, CA, USA). The sequences of the primers for ezrin were as follows: forward 5′-GTG GGA TGC TCA AAG ATA ATG C-3; reverse 5′-CAC CTC GAT GGT GTC AGG CT-3′. Real-time PCR was performed using an Mx3000p system (Stratagene, La Jolla, CA, USA) and all samples were run in triplicate. The quantification of gene expression levels was calculated relative to β-actin.

### Electrophoretic mobility shift assays (EMSAs)

NF-κB DNA-binding activity was measured by EMSA using nuclear extracts prepared from A549 cells. First, cells were scraped into 1 mL of PBS and centrifuged. The pelleted cells were then homogenized in buffer A [10 mmol/L HEPES (pH 7.9), 10 mmol/L KCl, 1.5 mmol/L MgCl_2_, 0.5 mmol/L dithiothreitol (DTT), 0.2 mmol/L PMSF, 0.5% NP-40], incubated on ice for 15 min, and then centrifuged for 5 min. Nuclear proteins were extracted by gently resuspending the nuclei pellet in buffer C [20 mmol/L HEPES (pH 7.9), 10 mmol/L KCl, 1.5 mmol/L MgCl_2_, 10% glycerol, 0.2 mmol/L EDTA, 0.5 mmol/L DTT, 0.2 mmol/L PMSF] together with buffer D (as for buffer C except 400 mmol/L KCl) added in a dropwise fashion. After 1 h incubation on ice, supernatants were collected by centrifugation at 13,800 × *g* for 15 min. An NF-κB specific oligonucleotide was end-labeled with [γ-^32^P] ATP using T4 polynucleotide kinase (New England Biolabs, Ipswich, MA, USA) and purified on a G-50 Sephadex spin column. Nuclear proteins were then incubated with ^32^P-labeled oligonucleotide for 30 min at room temperature. The DNA protein complexes were then resolved on a 4% non-denaturing polyacrylamide gel and subjected to autoradiography.

### CCK-8 assays

According to the manufacturer’s instructions, CCK-8 solution (Dojindo, Tokyo, Japan) was added to each well in a 96-well plate. Cells were incubated with Y-27632 (10 µmol/L or 50 µmol/L) for 4 h in a 37 °C, 5% CO_2_ incubator. The cells without Y-27632 treatment served as control. The OD value for each well was measured at 450 nm using a microplate reader.

### ELISA assays

Cell culture supernatants were collected and centrifuged at 1000 × *g* at 4 °C for 20 min, supernatants were then used to determine the levels of TNF-α, IL-1β, and HMGB1, in accordance with the manufacturer’s instructions (Elabscience, Wuhan, China). All samples were assayed in triplicate.

### Statistical analysis

All statistical analyses were performed using SigmaPlot 14.0 (Systat Software, Point Richmond, CA). Data are expressed as mean ± standard deviation (SD). Comparisons of two groups under the same treatment were performed by the Student's *t*-test. When statistical heterogeneity was evident, we applied Welch’s test. Significance was established at *p* < 0.05.

## Results

### LPS induced ezrin phosphorylation in A549 and HPAEpiC cells

**Fig. 1:**
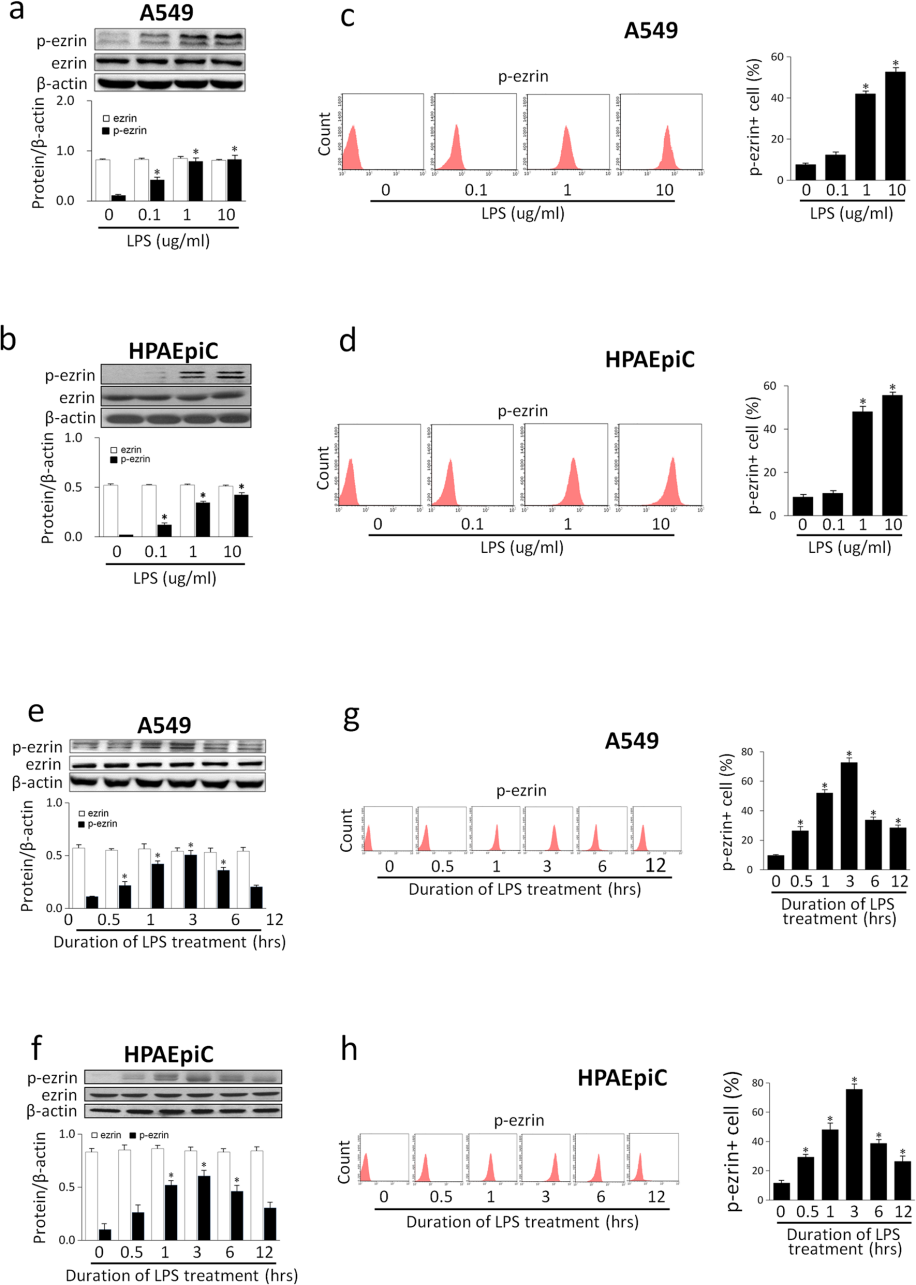
LPS induced ezrin phosphorylation in a concentration- and time-dependent manner. A549 and HPAEpiC cells were treated with LPS for 3 h at concentrations of 0 µg/mL, 0.1 µg/mL, 1 µg/mL, and 10 µg/mL, respectively. Phosphorylated ezrin protein level was evaluated by western blotting (**a**, **b**) and flow cytometry (**c**, **d**). A549 and HPAEpiC cells were treated with LPS (1 µg/mL) for 0 h, 0.5 h, 1 h, 3 h, 6 h and 12 h, respectively. Phosphorylated ezrin protein level was evaluated by western blotting (**e**, **f**) and flow cytometry (**g**, **h**). Data are expressed as means ± SD of triplicate samples. *p < 0.05 versus 0 µg/mL group

A549 and HPAEpiC cells were treated with LPS at different concentrations and time intervals. P-ezrin protein level was evaluated by western blotting and flow cytometry. Dose–response results indicated that a significant increase in LPS-induced ezrin protein phosphorylation was detected in the 1 µg/mL and 10 µg/mL groups as compared to the 0 µg/mL group (Fig. 1a–d). Western blotting and flow cytometry results showed that phosphorylated ezrin protein was enhanced in a time-dependent manner induced by LPS, which was elevated after 0.5 h, and reached a peak by 3 h and returned to baseline after 12 h. The expression level of ezrin protein remained unchanged (Fig. 1e–h).

### LPS activated RhoA/ROCK in A549 and HPAEpiC cells

**Fig. 2:**
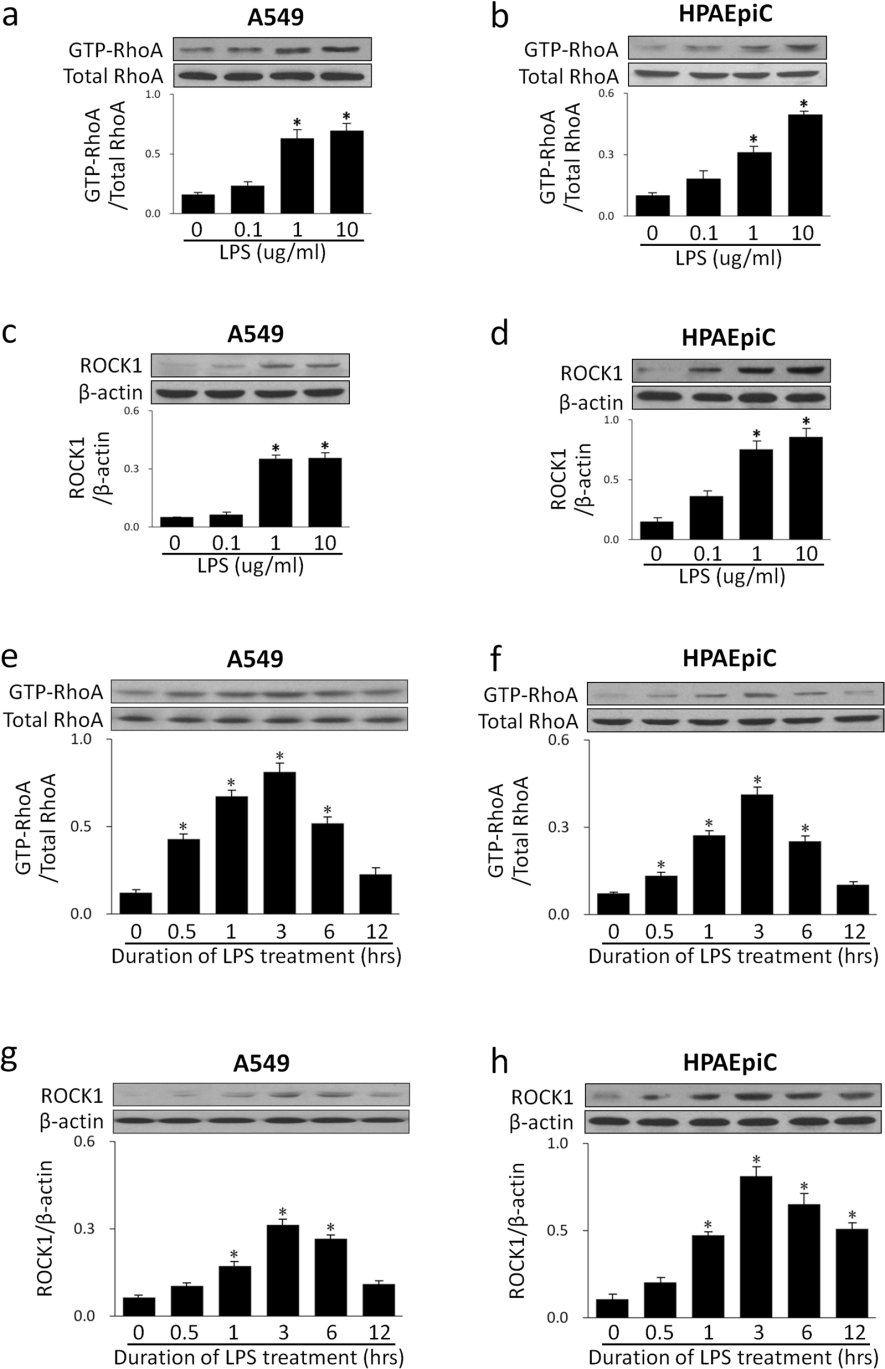
LPS up-regulated RhoA activity and ROCK expression in a concentration- and time-dependent manner. A549 and HPAEpiC cells were treated with LPS for 3 h at concentrations of 0 µg/mL, 0.1 µg/mL, 1 µg/mL, and 10 µg/mL, respectively. The activity of GTP-RhoA was evaluated by RhoA activity assays (**a**, **b**) and the expression of ROCK1 protein was examined by western blotting (**c**, **d**). A549 and HPAEpiC cells were treated with LPS (1 µg/mL) for 0 h, 0.5 h, 1 h, 3 h, 6 h and 12 h, respectively. The activity of GTP-RhoA was evaluated by RhoA activity assays (**e**, **f**) and the expression of ROCK1 protein was examined by western blotting (**g**, **h**). Data are expressed as means ± SD of triplicate samples. *p < 0.05 versus 0 µg/mL group

To investigate the potential involvement of the RhoA/ROCK signaling pathway in LPS-induced intracellular events, we examined the activity of GTP-RhoA and the expression of ROCK protein using RhoA activity assays and western blotting, respectively. Dose–response results indicated that an increase in LPS-induced RhoA activity and ROCK1 protein level were detected in the 1 µg/mL and 10 µg/mL groups as compared to the 0 µg/mL group (Fig. 2a, b, c, d). The time-response results showed that RhoA activity and ROCK1 protein level were up-regulated in a time-dependent manner induced by LPS, which were elevated after 0.5 h, and reached a peak by 3 h and returned to baseline after 12 h (Fig. 2e, f, g, h).

### ROCK mediates LPS-induced ezrin phosphorylation and relocation

**Fig. 3:**
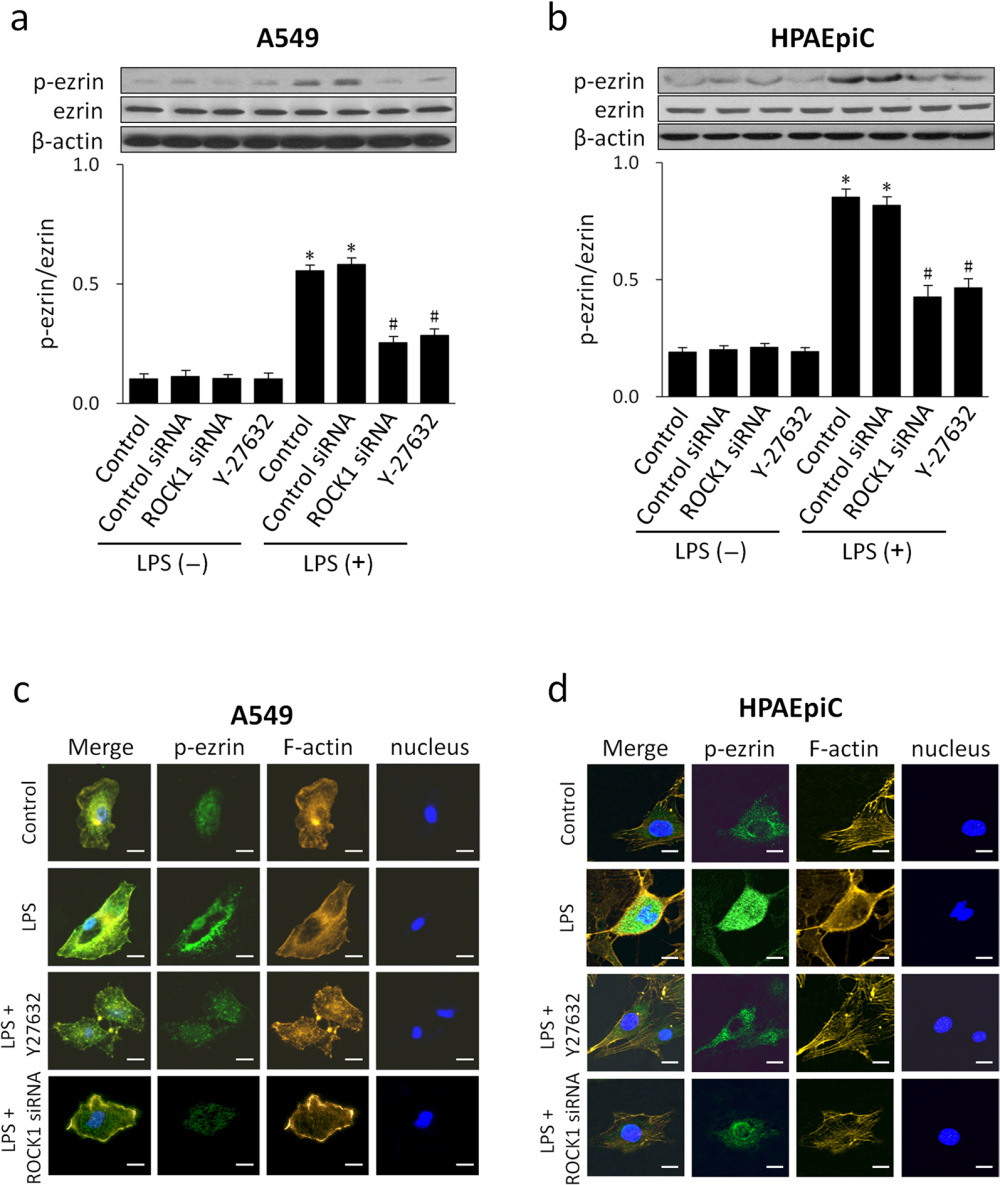
ROCK mediated LPS-induced ezrin phosphorylation and translocation. A549 and HPAEpiC cells were challenged with LPS (1 µg/mL) in the presence or absence of ROCK siRNA or Y-27632. Total ezrin and p-ezrin protein levels were detected by western blotting using β-actin as an internal reference (**a**, **b**). Data are expressed as means ± SD of triplicate samples. *p < 0.05 versus control, #*p* < 0.05 versus LPS. The intracellular localization of p-ezrin in resting and LPS-activated cells was investigated by immunofluorescence. P-ezrin was stained with Alexa Fluor 488-conjugated IgG (green), F-actin was stained with Rhodamine-phalloidin (yellow), and nuclei were stained with DAPI (blue) (**c**, **d**). Qualitative analysis was performed by confocal microscopy. Scale bar, 10 μm. All experiments were performed in three independent experiments

To investigate the involvement of ROCK in LPS-induced ezrin activation, A549 and HPAEpiC cells were pretreated with Y-27632 or transfected with ROCK1 siRNA before LPS stimulation. The expression levels of p-ezrin increased significantly in LPS-treated cells; pretreatment with Y-27632 or transfected with ROCK1 siRNA markedly inhibited the upregulation of p-ezrin (Fig. 3a, b). The intracellular localization of ezrin was next investigated by immunofluorescence. Resting cells exhibited weak green staining for p-ezrin that was mostly localized in the cytoplasm. Interestingly, p-ezrin appeared to translocate from the cytoplasm to the cell membrane in response to LPS, as evidenced by green punctuate staining; this was further indicated by co-localization with the reorganization of the F-actin cytoskeleton. This phenomenon was significantly abrogated by pre-treatment with Y-27632 or transfected with ROCK1 siRNA (Fig. 3c, d).

### Ezrin associated with MyD88/IRAK1 and Syk following LPS stimulation

**Fig. 4:**
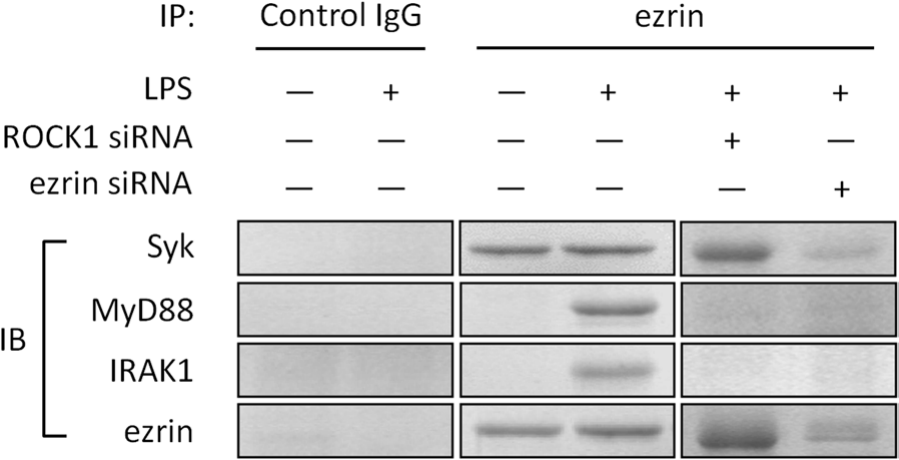
The association between ezrin, MyD88/IRAK1, and Syk. A549 cells were challenged with LPS (1 µg/mL) in the presence or absence of ROCK siRNA or ezrin siRNA. Lysates isolated from A549 cells exposed to LPS (+) or without LPS (−) were immunoprecipitated in the presence of anti-ezrin or control IgG antibody, followed by western blotting with anti-Syk, anti-MyD88, or anti-IRAK1 antibodies, respectively. IP: immunoprecipitation, IB: immunoblotting

We next evaluated potential interactions between ezrin and MyD88/IRAK1 or Syk. Following the immunoprecipitation of ezrin, western blotting of precipitated fractions showed that ezrin and Syk were present in both control and LPS-stimulated A549 cells; however, the interaction between ezrin and MyD88/IRAK-1 only occurred in LPS-stimulated samples. Transfection with ROCK1 siRNA or ezrin siRNA inhibited the interaction between ezrin and MyD88/IRAK-1. These results indicate that ezrin interacts with Syk in a constitutive manner, but associates with MyD88/IRAK1 in an LPS stimulation-dependent manner (Fig. 4).

### The suppression of ezrin inhibited the LPS-induced activation of p38 and NF-κB

**Fig. 5:**
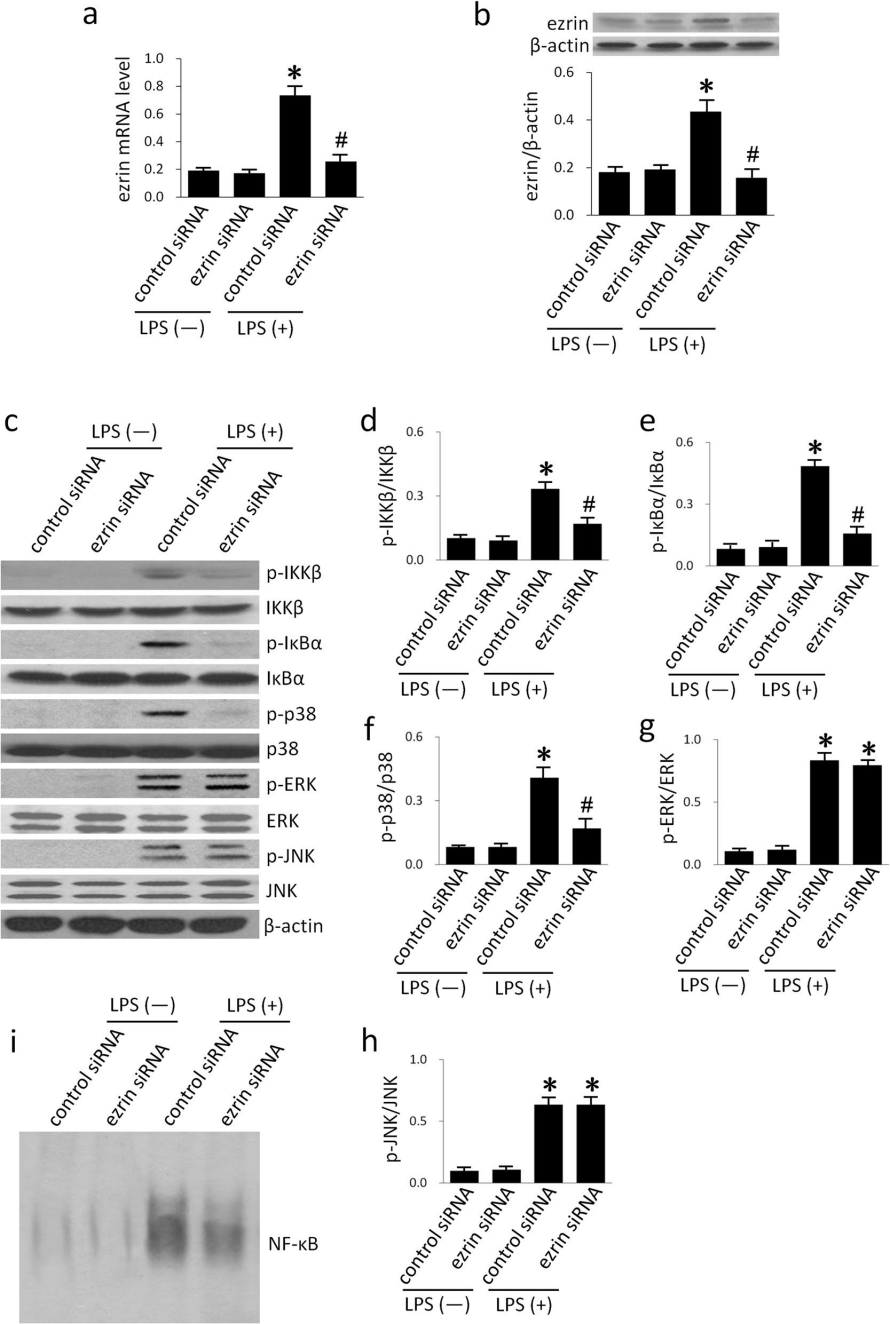
The suppression of ezrin inhibited LPS-induced activation of p38 and NF-κB. A549 cells were transfected with ezrin siRNA or control siRNA. The expression of ezrin mRNA was detected by qRT-PCR (**a**) and the protein level of ezrin was measured by western blotting (**b**). The effect of ezrin siRNA transfection on IKK, IκBα, and MAPKs activation was detected by western blotting (**c**–**h**), and NF-κB activation was determined by EMSA (i). Data are expressed as mean ± SD of triplicate samples. *p < 0.05 versus LPS(-) control siRNA group, #*p* < 0.05 versus LPS(+) control siRNA group

To explore ezrin-mediated downstream signaling, A549 cells were treated with ezrin-siRNA and then exposed to LPS. The suppression of ezrin mRNA and protein expression was detected by qRT-PCR and western blotting, respectively. The transfection of ezrin siRNA led to a marked reduction of ezrin levels (Fig. 5a, b). It has been shown that ERM participates in the NF-κB and MAPKs signaling pathways [13–15]. Therefore, we next investigated the potential involvement of p38, ERK1/2, JNK, IKK, IκBα, and NF-κB, in ezrin signaling. The phosphorylation of IKK, IκBα, p38, ERK, and JNK, as carried out by western blotting, revealed a marked increase in LPS-treated cells that were transfected with control siRNA (Fig. 5c–h). Similarly, the activation of NF-κB, as determined by EMSA, was increased in cells that were transfected with control siRNA and exposed to LPS (Fig. 5i). However, the suppression of ezrin partially suppressed LPS-induced NF-κB and p38 activation (Fig. 5c-f) but not ERK1/2 and JNK phosphorylation (Fig. 5c, g, h), thus suggesting that ezrin lies upstream of the NF-κB and p38 signaling pathways under LPS condition.

### The ROCK-ezrin pathway mediated the LPS-induced production of cytokines

**Fig. 6:**
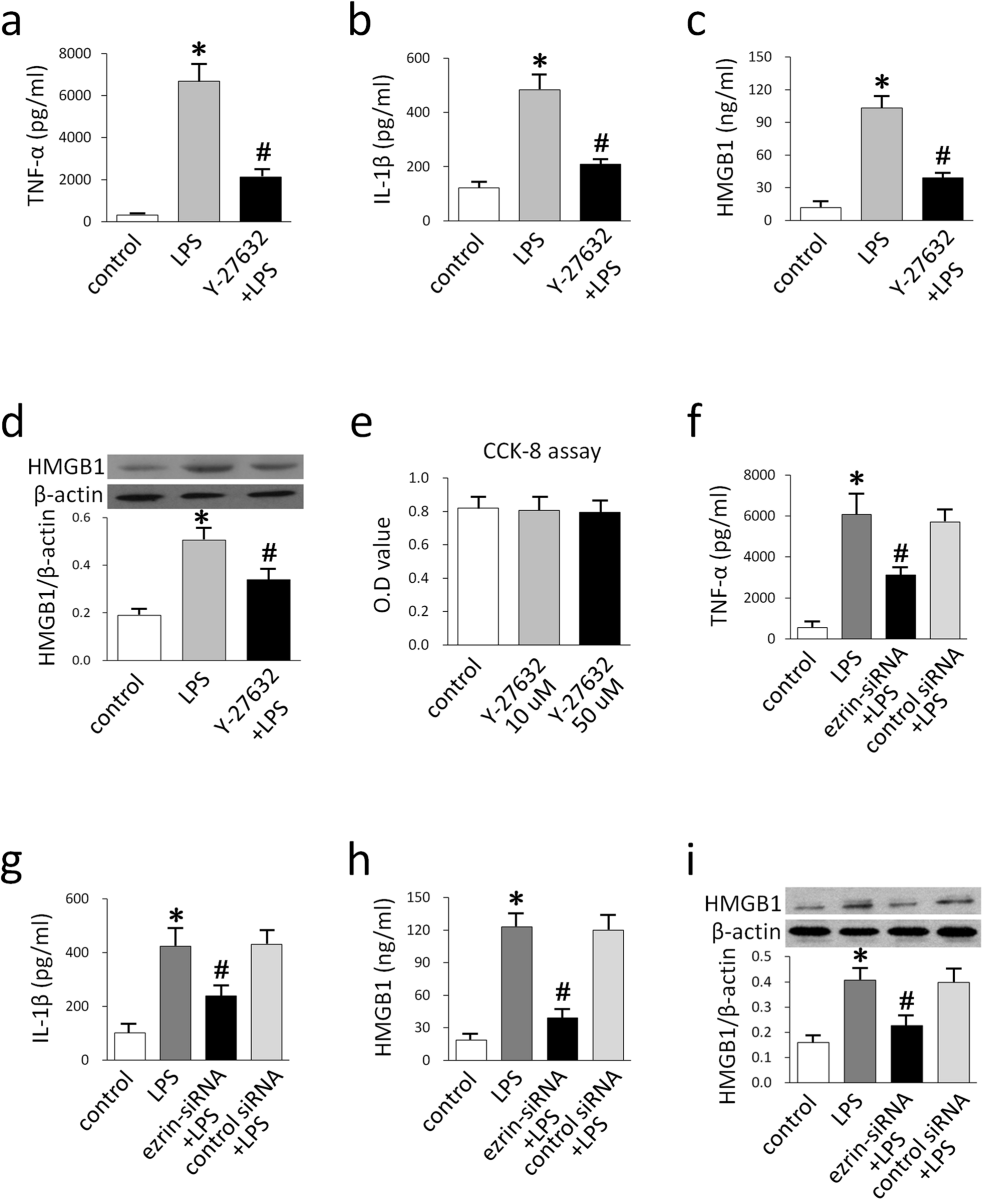
The suppression of ezrin inhibited the LPS-induced production of cytokines. A549 cells were pre-treated with Y-27632 and subjected to LPS. The release of TNF-α, IL-1β, and HMGB1 into the supernatants was then measured by ELISA (**a**–**c**). The expression level of HMGB1 in cell lysates was measured by western blotting (**d**). Cell viability was examined by CCK-8 assay (**e**). A549 cells were transfected with ezrin-specific siRNA; TNF-α, and IL-1β expression was measured by ELISA (**f**–**h**), and the cellular levels of HMGB1 was measured by western blotting (**i**). Data are expressed as mean ± SD of triplicate samples. *p < 0.05 versus control group, #*p* < 0.05 versus LPS group

To determine the functional relevance of ROCK-ezrin signaling in LPS-induced inflammatory responses, we examined the effect of suppressing ROCK-ezrin activation on the production of pro-inflammatory cytokines in A549 cells. As expected, LPS induced a significant increase in the release of TNF-α, IL-1β, and HMGB1 (Fig. 6a–c), and the cellular levels of HMGB1 (Fig. 6d). This up-regulation was inhibited by Y-27632 pre-treatment (Fig. 6a–d). CCK-8 assays showed that Y-27632 was not cytotoxic at the concentrations tested herein (Fig. 6e). As expected, LPS induced an increase in TNF-α, IL-1β, and HMGB1 in the cell culture supernatant (Fig. 6f–h), and the cellular levels of HMGB1 (Fig. 6i). Transfection with ezrin siRNA dramatically inhibited LPS-induced increases in the production of TNF-α, IL-1β, and HMGB1 (Fig. 6f–i).

## Discussion

ALI and its severe form, ARDS, are potentially fatal complications of sepsis and important causes of high mortality in critically ill patients [16]. Several recent studies have linked ERM to LPS induced lung injury [7, 8, 17]. However, the specific role of ezrin during this process has yet to be elucidated. In the present study, we demonstrate that LPS induced the phosphorylation of ezrin and the activation of RhoA/ROCK in a concentration- and time- dependent manner. The blockade of the RhoA/ROCK signaling pathway inhibited LPS induced ezrin phosphorylation and its translocation from cytoplasm to cell membrane. In addition, immunoprecipitation assays indicated that ezrin interacted with Syk in a constitutive manner, but only associated with MyD88/IRAK1 under LPS challenge. Ezrin also participates in p38 and NF-κB activation, but not ERK and JNK activation. Importantly, the knockdown of ezrin or the blockade of ROCK showed that the ROCK-ezrin pathway contributes to the upregulation and release of TNF-α, IL-1β and HMGB1 in response to LPS. Our findings provide evidence that ezrin is upregulated in a RhoA/ROCK-dependent manner by LPS and that ezrin acts as an upstream regulator of p38 and NF-κB activation and the production of pro-inflammatory cytokines.

While human alveolar epithelial cells may not completely recapitulate the pulmonary disorder in ALI/ARDS due to the complex nature of this phenomenon, the exposure of epithelial cells to LPS is well known to play a central role in the pathogenesis of acute lung inflammatory diseases [18]. Numerous experimental studies have used human A549 and HPAEpiC cell lines to examine the inflammatory response induced by LPS, as an acceptable, validated, and suitable in vitro airway epithelial injury model based on the initial steps of the development of ALI/ARDS [18–22]. Lung epithelial cells generate cytokines, chemokines, and other inflammatory peptides, in response to inflammatory stimuli; these factors are important determinants of the severity of the lung condition [23, 24]. As such, our cell culture model using LPS-induced A549 and HPAEpiC cells to examine pulmonary epithelial cytokines production provides relevant insight in the context of ALI/ARDS.

As a member of the ERM protein family, ezrin is considered not only as a cross-linker between the cytoskeleton and plasma membrane, but also an important signal transducer that participates in cell adhesion and motility [25]. For these cellular functions, ezrin needs to be activated. When subjected to stimuli, ezrin binds to phosphatidylinositol 4,5-bisphosphate (PIP2) in the cell membrane and subsequently the threonine residue (ezrin^T567^) at the C-terminal is phosphorylated, thus leading to dissociation between the N-terminal and C-terminal domains; this unmasks binding sites for other molecules [26]. ERM is phosphorylated by TNF-α, advanced glycation end products (AGEs), 2-Methoxyestradiol, and thrombin, and are known to modulate endothelial hyperpermeability [27–30]. Here, we report the phosphorylation of ezrin induced by LPS, as well as its translocation from the cytoplasm to the cell membrane in manner that is concomitant with the reorganization of the F-actin cytoskeleton. It has been shown that ERM is translocated to the plasma membrane as a result of interactions with the cytoplasmic domain of integral membrane proteins, such as CD44, thus providing a site for association with actin filaments [31]. Thus, LPS-induced ezrin phosphorylation was accompanied by cytoskeleton reorganization, indicating that LPS-induced conserved threonine residue phosphorylation might contribute to the formation of actin filaments and the reorganization of the actin cytoskeleton. These results are consistent with previous findings in pulmonary endothelial cells that were stimulated by TNF-α [27], 2-Methoxyestradiol [28], and thrombin [30], thus suggesting that the activation of ezrin is part of a general response to inflammatory stress [32].

Several protein kinases, including Rho/ROCK and PKC, are known to phosphorylate ERM on the C-terminal threonine [33, 34]. ERM can be directly phosphorylated by ROCK to enhance its binding to membrane proteins and F-actin, thus regulating the reorganization of actin filaments to participate in a range of cellular functions [35, 36]. ROCK is a major downstream effector for Rho. The expression of ROCK1 has been detected in the lungs, liver, kidneys, spleen, and testes, while ROCK2 is predominantly expressed in the heart, muscle, and brain [37]. To determine whether Rho/ROCK1-mediated LPS induced the phosphorylation of ezrin, we pretreated cells with ROCK1 siRNA or the ROCK1 inhibitor Y-27632 prior to LPS stimulation. Results showed that the blockade of ROCK1 prevented LPS-induced ezrin phosphorylation and its subsequent translocation from the cytosol to the cell membrane, as well as F-actin reorganization, suggesting a critical role for ROCK in the activation of ezrin. Interestingly, ERM can play a dual role in the Rho/ROCK signaling pathway by acting both upstream and downstream of ROCK [35]. ERMs can be activated by ROCK; once activated, ERMs dissociate Rho-GDI (a GDP dissociation inhibitor) from Rho and thereby activates Rho/ROCK [36]. Thus, the rapid activation of ezrin and Rho/ROCK observed in the present study suggests that the levels of phosphorylated ezrin may act as a limiting factor for signaling pathways involving ezrin and that the marked upregulation of ezrin in response to LPS could be an important feed-forward mechanism.

Interestingly, Feng GE et al. [38] recently reported that the EphA2 receptor plays an essential role in LPS-induced ALI and EphA2 antagonism and has protective effects against LPS-induced ALI via the Nrf2/HO-1, TLR4/MyD88 and RhoA/ROCK pathways. The involvement of RhoA/ROCK has also been reported in TLR4 signaling pathways. For example, LPS rapidly activated RhoA and subsequently increased the transcription of IL-8 in human peripheral blood monocytes by acting on TLR4 [39]. P120 selectively regulates LPS/TLR4 signaling via RhoA-mediated TLR4 endocytosis in macrophages [40]. Moreover, the inhibition of TLR4 expression significantly reduced LPS-induced RhoA activities in vascular endothelial cells [41]. Thus, evidence suggests that RhoA/ROCK may act as a “molecular switch” in the activation of TLR4-mediated innate immune responses [42]. However, the mechanisms involved in the crosstalk between the RhoA/ROCK and TLR4 pathways after LPS challenge remains unclear.

It has been shown that the phosphorylation of ERM is critical for recruiting Syk, a key molecule in signaling processes that is initiated by pattern recognition receptors (PRRs) [43, 44]. Moreover, macrophages that were pre-incubated with moesin antibody exhibited total inhibition of IRAK phosphorylation and its association with MyD88/IRAK in response to LPS [45]. Thus, it appears that ERM acts as an adapter protein that links Syk to MyD88/IRAK. However, whether ezrin interacts with Syk and/or MyD88/IRAK1 in the presence of LPS has never been investigated. Here, the potential association between either Syk or MyD88/IRAK1 and ezrin in lung epithelial cells was evaluated by co-immunoprecipitation. We found that the expression of Syk corresponded with that of ezrin, indicating that Syk interacts with ezrin in a constitutive manner. However, MyD88/IRAK1 immunoprecipitated with ezrin only under LPS challenge. The results demonstrate that ezrin interacts with MyD88/IRAK1 in a LPS stimulation-dependent manner and suggest that ezrin links Syk to MyD88/IRAK1 following LPS stimulation. Collectively, our results suggest that ezrin, and its interaction with Syk and MyD88/IRAK1, can initiate Rho/ROCK-ezrin/Syk-MyD88/IRAK1 signaling and act as an adapter molecular that links the RhoA/ROCK and TLR4 pathways in response to LPS.

It has previously been shown that ERM contributes to the LPS-induced production of cytokines [9, 45, 46]. To further explore the functional relevance of the ROCK-ezrin pathway in LPS- induced responses, we used Y-27632 to inhibit ROCK activity and siRNA to suppress ezrin, and found that LPS induced significant reductions in the production of TNF-α, IL-1β, and HMGB1. The observed reduction in cytokine production was associated with the inhibition of NF-κB and p38 MAPK activation. Interestingly, Zawawi et al.[45] previously reported that the blockade of moesin function inhibited the LPS-induced activation of MyD88, IRAK and TRAF6, as well as subsequent MAPK activation and NF-κB translocation to the nucleus. Weng et al. [47] also confirmed that the phosphorylation of ezrin triggered MAPK signal transduction in tumor progression. Thus, our results are consistent with a specific role for the ERM family in the activation of p38 and NF-κB activation.

There are two major limitations in this study that could be addressed in future research. First, although two types of human pulmonary alveolar epithelial cell lines, A549 and HPAEpiC, were employed in the present study, given the heterogeneity and variability of their potential roles, other surrounding cells may also play influential roles. Consequently, alveolar epithelial cells may not completely replicate the pulmonary disorder in ALI/ARDS due to the complex nature of this phenomenon. Second, it should also be noted that observation of cell ultrastructure with transmission electron microscopy may better reveal the cell morphological changes under different conditions, including LPS challenge. Therefore, more comprehensive, and in-depth research in the future will be important to figure out the complex regulatory mechanisms and signal pathways involved in ALI/ARDS,

## Conclusions

**Fig. 7:**
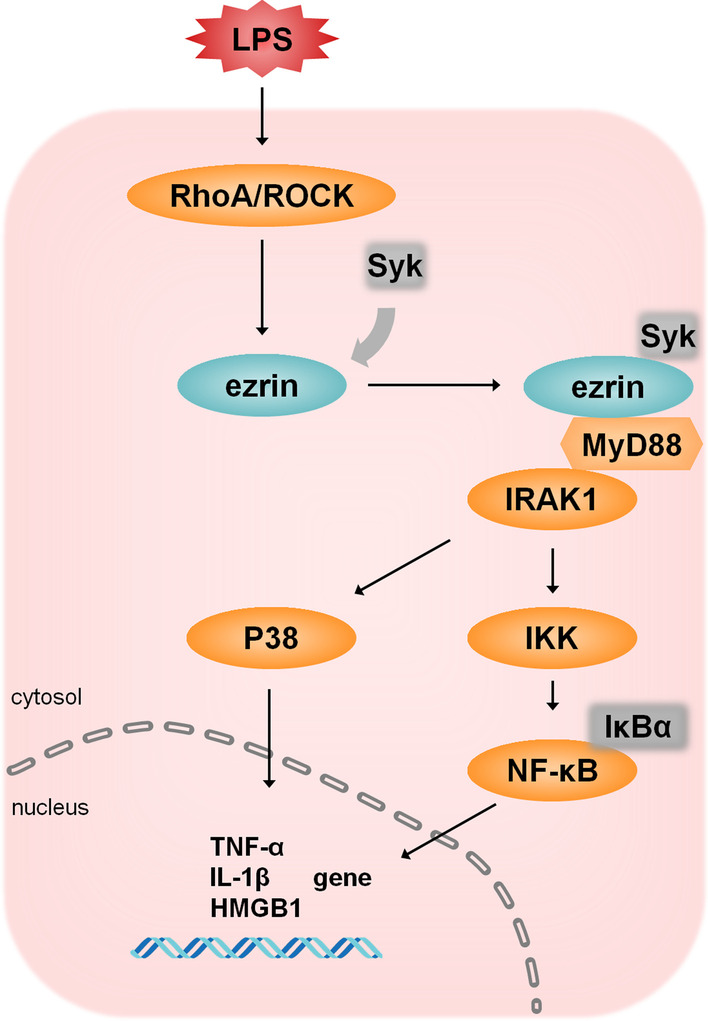
A schematic summary of the ROCK-ezrin pathway in response to LPSs

Data presented in this manuscript provide novel insights into the molecular mechanisms and signaling pathways in the response of alveolar epithelial cells to LPS. Based upon these findings, we suggest a model for LPS signaling events (Fig. 7). In this model, LPS activates RhoA/ROCK and subsequently induces the phosphorylation of ezrin; this process requires the assistance of Syk. Phosphorylated ezrin then translocates from the cytoplasm to the plasma membrane where it recruits IRAK1 with the assistance of MyD88. The four-molecule cluster (ezrin, Syk, MyD88 and IRAK1) then induces activation of the NF-κB and p38 pathways and ultimately enhances the gene expression of inflammatory cytokines.

## Data Availability

All data generated in this study are available from corresponding author on reasonable request.

## Abbreviations

ALI: Acute lung injury
ARDS: Acute respiratory distress syndrome
BCA: Bicinchoninic acid
BSA: Bovine serum albumin
EMSAs: Electrophoretic mobility shift assays
ERM: Ezrin/radixin/moesin protein
HMGB1: High mobility group box 1 protein
IL-1β: Interleukin-1β
IRAK-1: IL-1R-associated kinase 1
LPS: Lipopolysaccharide
MAPK: Mitogen-activated protein kinase
MyD88: Myeloid differentiation factor 88
NF-κB: Nuclear factor-κB
PBS: Phosphate buffered saline
PIC: Protease inhibitor cocktail
PMSF: Phenylmethylsulfonyl fluoride
PVDF: Polyvinyl difluoride
RIPA: Radio immunoprecipitation assay
ROCK: Rho-associated coiled-coil containing protein kinase
SD: Standard deviation
SDS-PAGE: Sodium dodecyl sulfate polyacrylamide gel electrophoresis
TBST: Tris-buffered saline containing Tween 20
TLR4: Toll-like receptor 4
TNF-α: Tumor necrosis factor-α
